# Enhancing genetic management in captive Asian elephants: Evaluation of mitochondrial single-nucleotide polymorphism markers for improved breeding and conservation in the Elephant Kingdom, Thailand

**DOI:** 10.14202/vetworld.2025.565-572

**Published:** 2025-03-09

**Authors:** Chavin Chaisongkram, Nuttapon Bangkaew, Bunnada Siriporn, Krittika Por-armart, Premika Charoenchai, Nunnapat Mahaveero, Tarid Purisotayo

**Affiliations:** 1 Khon Kaen Zoo, Zoological Park Organization of Thailand, Khon Kaen, 40280, Thailand; 2 Elephant Kingdom, Zoological Park Organization of Thailand, Surin, 32120, Thailand; 3 Faculty of Veterinary Science, Mahasarakham University, Maha Sarakham, 44000, Thailand; 4 Veterinary Infectious Disease Research Unit, Mahasarakham University, Maha Sarakham, 44000, Thailand

**Keywords:** Asian elephants, conservation breeding, genetic diversity, kinship coefficients, molecular markers, pedigree analysis

## Abstract

**Background and Aim:**

Maintaining genetic diversity and preventing inbreeding depression in captive Asian elephants (*Elephas maximus*) are crucial challenges that require effective breeding management and conservation strategies. This study aimed to assess genetic diversity and evaluate the effectiveness of currently available molecular markers as breeding management tools in captive Asian elephant populations at the Elephant Kingdom (EK) in Thailand.

**Materials and Methods:**

Data were collected from identification certificates of elephants at the EK, including age, sex, parentage, and genotypes of 16 mitochondrial single-nucleotide polymorphisms (mtSNPs). An observation-based pedigree was constructed to estimate pedigree-based kinship coefficients, which were compared to molecular-based kinship coefficients. Population and genetic diversity indices were analyzed. Pedigree-based and molecular-based kinship coefficients were compared to evaluate marker efficiency.

**Results:**

The population had a balanced sex ratio of 0.97:1 (male: female). Based on the 16 mtSNPs, the mean observed heterozygosity and expected heterozygosity were 0.4451 and 0.5278, respectively, indicating a heterozygous deficit. The pedigree-based and molecular-based kinship coefficients differed significantly and negatively correlated (*r* = −0.28, p < 0.05). The molecular-based method estimated higher kinship coefficients than the pedigree-based method.

**Conclusion:**

Evaluation of mtSNP markers highlights their utility in assessing genetic diversity and kinship in captive Asian elephant populations in EK, Thailand. However, the observed discrepancies between pedigree-based and molecular-based kinship estimates underscore the limitations of the current mtSNP panel. The findings emphasize the need for integrating nuclear SNPs to enhance the precision of genetic management strategies, enabling better-informed decisions to preserve genetic diversity and mitigate inbreeding risks in conservation breeding programs, not only for the EK but also as a framework that can be adapted for broader conservation efforts.

## INTRODUCTION

Managing small wildlife populations involves implementing strategies to ensure survival and genetic diversity. Conservation efforts typically focus on habitat protection, controlling factors that contribute to population decline, and promoting breeding programs. Breeding programs, both in captivity and in the wild, are essential for increasing numbers and maintaining genetic diversity, which prevents inbreeding and boosts the population’s resilience to catastrophic changes [1]. Effective breeding and genetic management require continuous monitoring and research to adapt to strategies as needed. Molecular markers play a crucial role in these processes. However, molecular markers for non-domestic species are often limited. In Thailand, where domestic Asian elephants (*Elephas maximus*) are integral to culture and economy, their roles have evolved over centuries, from warfare and logging to tourism and conservation. Logging bans in the late 20^th^ century displaced many elephants, leading to their adoption in tourism and performance activities [2]. Despite regulations like the Wildlife Preservation and Protection Act B.E. 2535 (amended in 2019), which prohibit exploitative practices such as street begging, challenges remain in managing their welfare and genetic diversity [3]. A recent census in 2020 estimated ~3,800 domestic elephants in Thailand, emphasizing the urgent need for effective breeding management strategies to sustain this population [4]. To address these challenges, the Zoological Park Organization of Thailand established the Elephant Kingdom (EK) in 2010. Located in northeast Thailand, EK provides sanctuary to approximately 200 elephants, provides financial and veterinary support to owners, and promotes structured breeding programs.

Although the establishment of the EK represents a significant step toward addressing welfare and management challenges, its breeding practices reveal underlying issues related to genetic diversity and resilience. Breeding management in EK is hindered by limited candidate diversity, with bulls often selected based on temperament and physical traits. This practice is associated with the risk of inbreeding, genetic diversity reduction, and population resilience to diseases and environmental changes. Although captive Asian elephants are critical for conservation, genomic data for these species are insufficient for comprehensive studies. Previous genetic assessments have used microsatellites [5–7]. However, we chose to use single nucleotide polymorphism (SNP) markers over microsatellites because SNPs are high-throughput, more reproducible, and easier to standardize across laboratories compared with microsatellites [8]. These are particularly important for long-term monitoring and management of captive populations, where consistent and comparable data are crucial. We selected these mitochondrial SNPs (mtSNPs) due to their immediate availability and the established protocols for elephants in Thailand. While microsatellites have been widely used in elephant genetics, the increasing availability of SNP data for elephants [9, 10] provides an opportunity to evaluate their efficacy in population management [11]. However, we acknowledge that the use of mtSNPs alone has limitations because they are maternally inherited, which may limit their utility for comprehensive inbreeding analyses and may underrepresent the complexity of genetic relationships within the population. Despite these limitations, mtSNPs have high mutation rates [12] that effectively capture recent evolutionary events. The ease of amplification and sequencing makes them practical for studies in non-model or endangered species, where nuclear data may be challenging to obtain. Inbreeding within a population refers to the mating of genetically related individuals (i.e., descendants from a shared ancestor), which can lead to a higher probability of an offspring inheriting identical and/or harmful alleles from its parents [13]. The effects of inbreeding, such as its negative effects on fertility and growth rate, have been widely addressed and monitored in domestic animals [14, 15]. However, assessing the impact of inbreeding in captive non-model or wild species poses significant challenges. Factors such as limited genetic data, small sample sizes, complex population dynamics, and varying degrees of inbreeding hinder comprehensive studies [16].

Most importantly, non-model species often lack molecular markers for this purpose. Domestic elephants legally require identification certificates. Initially, these certificates included details such as the elephant’s birth date, registration date, place of registration, names of the elephant and its owner, parents’ names, sex, number of toes, tusk characteristics, back shapes, and other distinct markings. Subsequently, the certificates were updated to incorporate microchip numbers and DNA markers for enhanced identification [10]. These markers were originally developed for forensic purposes to differentiate between ivories obtained from Asian and African elephants. Although these markers have been used primarily for individual identification in captive Asian elephant populations, their efficiency in supporting effective breeding management has not yet been thoroughly assessed.

This study aimed to assess genetic diversity and specifically evaluate the effectiveness of the currently used SNP set as a tool for breeding management within the context of its existing application. An observation-based pedigree was constructed to estimate pedigree-estimated kinship coefficients, which were subsequently compared to molecular-estimated kinship coefficients. A comparison of the two methods would allow for the assessment of marker efficiency and provide valuable insights into the potential advantages and limitations of each approach. Kinship coefficients were used to demonstrate their application in breeding management. To the best of our knowledge, there is only one study [17] that examined how pedigree-based kinship coefficients compare to those estimated from mtSNP data in Asian elephants. This gap is particularly evident in structured captive breeding programs like EK, where the efficacy of molecular markers in accurately representing kinship remains largely unexplored. By directly comparing pedigree- and mtSNP-based kinship estimates within this context, our study addresses a critical knowledge gap in elephant population genetics, offering insights with the potential to enhance breeding strategies and genetic management practices.

## MATERIALS AND METHODS

### Ethical approval

This study involved only a retrospective analysis of existing identification certificates of elephants at the EK. No animals were handled, sampled, or subjected to any procedures for this research.

### Study period and location

The study was conducted from January to March 2022 at the EK, a sanctuary established in 2010 by the Zoological Park Organization of Thailand located in Surin province in northeastern Thailand.

### Data collection and population demography

Data collection was carried out in March 2022 due to the dynamic nature of the population, which necessitated a clear cutoff point to ensure data consistency and reliability. As a result, individuals born or enrolled in the EK after that time frame were not considered in this research. Information on the elephants was obtained from the identification certificates of the animals managed by the EK. This included age, sex, name of the elephant and its parents, and genotypes of 16 SNPs [9, 10]. Each elephant was assigned an identification number (ID). The sex ratio was defined as the ratio of male-to-female participants. To determine the age structure of the elephant population, animals were classified based on age [18]. This classification was performed by dividing them into five distinct categories: Calves and juveniles (<5 years of age), sub-adults (5–15 years), young adults (15–30 years), and old adults (more than 30 years). Sub-adults and young adults were considered pubertal elephants and potential breeding candidates [18].

### Statistical and genetic analyses

All statistical analyses were performed using R software version 4.2.2 (R Core Team, R Foundation for Statistical Computing, Vienna, Austria) [19] and GENEPOP version 4.7.5 (Laboratoire de Génétique et Environnement, Montpellier, France) [20]. Descriptive statistics were used to summarize population demographics and genetic diversity indices, including mean, standard deviation, and range. The Hardy–Weinberg equilibrium (HWE) for individual SNP loci was tested using an exact test with Bonferroni correction to account for multiple comparisons.

Observed (H_o_) and expected (H_e_) heterozygosity values were analyzed to evaluate deviations from genetic equilibrium. SNP loci that significantly deviated from HWE were excluded from further marker-based kinship coefficient analyses. SNP deviations from HWE can indicate underlying issues, such as genotyping errors, selection, or linkage disequilibrium [21], which may affect the reliability of these markers. In addition, marker-estimated kinship assumes that markers are in HWE, as deviations can lead to inaccurate estimates of relatedness and misinterpretation of genetic relationships [22]. All statistical tests were two-tailed, and results are reported with 95% confidence intervals where applicable.

Comparisons between pedigree-based kinship coefficients (K_ped_) and marker-based kinship coefficients (K_mark_) were assessed using the Wilcoxon signed-rank test [23] to determine statistical differences (p < 0.05), with the null hypothesis that the K_mark_ would not differ statistically from the K_ped_. Pearson’s correlation coefficient (r) was calculated to evaluate the relationship between K_ped_ and K_mark_, with statistical significance set at p < 0.05.

### Pedigree construction and kinship coefficient estimation

The kinship coefficient (K) measures genetic relatedness between individuals/populations, reflecting the probability that alleles at a locus are identical by descent [24]. Pedigree was considered a standard method for estimating the kinship coefficient to assess the efficiency of molecular markers currently available for Asian elephants because it allowed the assumption that shared alleles were derived by descent. We used data from the identification certificates of the elephants housed at the EK to develop the pedigree and estimate the kinship coefficients. The Kinship2 R package (Mayo Clinic, Rochester, MN, USA) [25] was used to estimate K_ped_. The kinship coefficient of an individual was calculated by averaging all its pairwise kinships with other elephants in the EK. Population K was defined as the average of all individual kinship groups within the population. Pubertal elephants that showed individual kinships below the population K_ped_ threshold were considered genetically valuable and deemed as potential breeding candidates [26]. Parentage contributions of individuals were determined based on the constructed pedigree to identify breeding individuals that could contribute to inbred elephants in the following generations. The K_mark_ values were estimated using the Popkin R package (Institute for Integrative Genomics, Princeton University, Princeton, NJ, USA) [27]. Only SNPs that did not deviate from the HWE were considered for the kinship estimations. The individual and population K_mark_ values were calculated similarly to those for K_ped_.

### Comparison of estimated kinship coefficients

The estimated population K_ped_ and population K_mark_ were compared to assess the efficiency of the molecular markers. Individuals without genotypic data were excluded from the study. The differences or similarities between the estimates obtained by the two methods would imply the efficiency of the currently available markers. The correlation between the estimates of an individual K_mark_ and K_ped_ was determined. The potential application of molecular markers as an alternative to pedigree analysis could demonstrate a positive correlation between the results obtained using the two techniques.

## RESULTS

### Population demography

**Figure 1:**
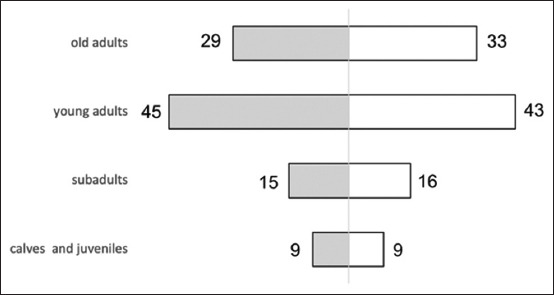
The demographic makeup of the population at Elephant Kingdom is illustrated by the age-sex structure. The shaded and white bars represent the numbers and proportions of male and female elephants, respectively.

The study included 199 elephants managed by the EK, comprising 98 males (49.3%) and 101 females (50.7%), resulting in a nearly balanced sex ratio of 0.97:1 (male: female). The population’s age structure revealed 18 calves and juveniles (<5 years), 31 sub-adults (5–15 years), 88 young adults (15–30 years), and 62 old adults (>30 years), indicating that most individuals were within the reproductive or post-reproductive age categories (Figure 1). The observed skew in age categories, particularly the predominance of older adults, has significant implications for breeding programs. Older individuals may experience decreased reproductive success due to age-related physiological limitations [28], which could hinder the sustainability of the population. In addition, this demographic imbalance might necessitate more intensive management efforts to ensure successful breeding and the inclusion of younger individuals to maintain genetic diversity and prevent inbreeding.

### Genetic diversity

Of the 199 elephants, genetic data from 176 individuals were analyzed (Table 1). Across the 16 mtSNP loci (Supplementary data), the mean observed heterozygosity (H_o_) was 0.4451 (range: 0.1932–0.5568), and the mean expected heterozygosity (H_e_) was 0.5278 (range: 0.4988–0.5896). The HWE test indicated significant deviations for two loci (SNP5 and SNP10, p < 0.05 after Bonferroni correction), which were excluded from further marker-based kinship analysis. The remaining 14 loci were retained for subsequent evaluations (Table 1).

**Table 1:** Global and per-locus genetic diversity of elephants under EK management.

Locus	N_a_	N	H_O_	H_e_	HWE
SNP1	2	175.0	0.4114	0.5143	0.0609
SNP2	2	176.0	0.5398	0.4999	0.3742
SNP3	2	176.0	0.4773	0.5245	1.0
SNP4	2	176.0	0.4659	0.4988	0.3749
SNP5	2	176.0	0.3807	0.5071	0.0029^a^
SNP6	2	176.0	0.4545	0.5079	0.354
SNP7	2	176.0	0.4205	0.5691	0.8631
SNP8	2	176.0	0.5568	0.5095	0.0896
SNP9	2	176.0	0.4886	0.5027	0.8789
SNP10	2	176.0	0.1932	0.5245	0.0^a^
SNP11	2	176.0	0.3807	0.5896	0.3607
SNP12	2	176.0	0.5114	0.5064	0.6473
SNP13	2	176.0	0.4489	0.5122	0.3618
SNP14	2	176.0	0.4602	0.5896	0.1379
SNP15	2	176.0	0.4432	0.5608	1.0
SNP16	2	176.0	0.4886	0.5271	0.7461
Global average	2	-	0.4451	0.5278	-

N_a_=Number of alleles per locus, N=Numbers of individuals with genotypic data, H_o_=Observed heterozygosity, H_e_=Expected heterozygosity, HWE=Hardy-Weinberg Equilibrium, EK=Elephant Kingdom, SNP=Single-nucleotide polymorphic.

a SNPs deviating from HWE

### Pedigree construction and kinship coefficients

**Figure 2:**
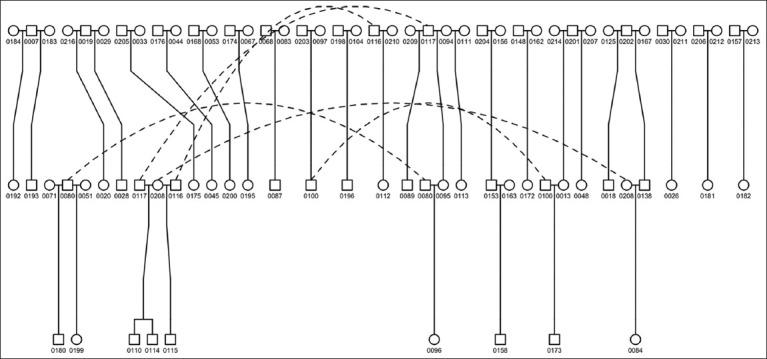
Pedigrees were constructed based on parentage information recorded in the identification certificates. Rectangles represent males and circles represent females. The identification numbers of the elephants were obtained using four-digit codes. The dotted lines link the same individuals to enhance the visualization of the pedigree.

A pedigree was constructed for 215 elephants, including 199 from the EK and 16 from neighboring villages. Kinship coefficients derived from the pedigree (K_ped_) showed a mean population value of 0.0032 (range: 0.0023–0.0090). Parentage analysis revealed that 29 dams and 22 sires contributed to 33 calves (Figure 2). Notably, seven sires fathered multiple calves, with one sire (ID 0117) producing 5 offspring (Supplementary data). Only two dams (ID 0208 and ID 0094) contributed to multiple calves (Supplementary data).

Marker-based kinship coefficients (K_mark_) were calculated for 176 individuals using the 14 mtSNP loci (Supplementary data). The mean population K_mark_ was 0.4201 (range: 0.2096–0.5661), which was significantly higher than the mean K_ped_ (Wilcoxon signed-ranks test, p < 0.001). A weak negative correlation between K_mark_ and K_ped_ was observed (r = −0.28, p < 0.05), indicating discrepancies between the molecular and pedigree-based estimates (Supplementary data).

### Kinship method comparison

The observed discrepancy between K_ped_ and K_mark_ likely reflects the limitations of the mtSNP panel, which was originally designed for wildlife forensics rather than population management. The higher K_mark_ values suggest that SNP markers overestimate genetic relatedness due to their conserved nature across elephant species. In contrast, K_ped_ estimates may be underestimated due to potential inaccuracies in founder assumptions and missing pedigree data.

## DISCUSSION

We determined the male-to-female ratio to be 0.97:1, which effectively indicated an equal distribution of males and females. Populations with skewed sex ratios, either males or females, were found to be at a higher risk of extinction due to the surplus of the other sex [29]. Skewed sex ratios of pubertal animals have been documented in many wild species, and the reasons behind such skewness vary among different species/orders of animals. In many wild bird populations, the adult sex ratio is biased, with males constituting roughly 33% more than females [30]. The primary factor contributing to this disparity was the higher mortality rate among females. Sea turtles with temperature-dependent sex determination can sustain populations through balanced sex ratios. However, if incubation temperatures rise significantly due to future global warming, the high mortality rate and production of only female hatchlings may put the species at risk [31]. The elephant population in the EK was not biased toward a particular sex ratio, but certain preferences toward specific breeding candidates were observed (IDs 0177, 0080), leading to potential inbreeding and a decrease in the genetic diversity of the population.

Analysis of the genetic diversity of the EK population revealed a deficit in heterozygosity based on the genotypes of the 16 mtSNPs. This finding is consistent with the observed negative correlation and disparity between the kinship coefficients derived using the two estimation methods. Specifically, we found that the K_mark_ value was significantly higher than the K_ped_ value. The differences observed between the two methods are consistent with Goudet *et al*. [32], who identified two primary factors contributing to mismatches between pedigree-based and marker-based kinship estimates: inaccuracies in pedigree records and variability in the genetic backgrounds of the founder population. Specifically, K_mark_ values may be inflated in scenarios where markers are conserved across species or exhibit low variability, as is likely the case with the SNP panel used in this study, which was originally designed for wildlife forensic purposes [9]. Given the main objective of marker design, these SNPs would be, to some extent, conserved across elephant species. This could lead to a heterozygosity deficit and a high marker-estimated kinship coefficient. Underestimation of the K_ped_ might also partially contribute to the observed disparity. Adult elephants who had been enrolled in the EK since the beginning of the program were considered the founders of the population for pedigree analysis purposes. This assumption was questionable based on personal communications with the owners of the elephants. There was a substantial likelihood that these founders were relatives. Some founders were born in Surin province and sold to other locations (e.g., as a begging elephant) before being repatriated to the EK. Most elephants in Northeastern Thailand originate from Surin Province, where the ethnic group known as the Suay has a long tradition of elephant ownership, often inheriting elephants through familial lines across generations. Furthermore, the frequent movement of elephants within the region, including their use in tourism and ceremonial activities, may lead to repeated interactions between individuals from the same familial lineage, compounding the genetic similarity within the population. Such practices are common in the region and may introduce underestimation of the K_ped_. This is consistent with findings in other captive elephant populations, suggesting a high probability of relatedness among founders [17].

For over two decades, microsatellite markers have been widely used in elephant genetics research, providing valuable insights into population structure, genetic diversity, and kinship relationships. For instance, Vidya *et al*. [33] employed microsatellites to investigate the genetic structure of Asian elephant populations across India, whereas Thitaram *et al*. [7] used them to assess the genetic diversity of captive Thai elephant populations. The high polymorphism of microsatellites allows for the fine-scale resolution of genetic relationships, which is crucial for the management of small captive populations. However, standardized panels of microsatellite markers for elephants are necessary to allow comparisons across studies and populations [34, 35]. Although our study focused on mtSNPs due to their immediate availability in our population, we acknowledge that incorporating microsatellite data could provide a more comprehensive understanding of the genetic diversity and kinship relationships within the EK population. However, the limitations of microsatellite markers have necessitated the need for nuclear SNPs to facilitate multiple-population genetic studies and collaboration between laboratories. Future research should incorporate nuclear SNP markers to complement mitochondrial (mt) data and provide a more comprehensive understanding of genetic diversity and kinship in captive Asian elephant populations. Nuclear SNPs, with their biparental inheritance and higher variability, can help address limitations in mt studies and yield more accurate kinship and relatedness estimates [36]. Broader genomic datasets, including whole-genome sequencing and population-wide sampling, should also be used to capture a more complete genetic landscape [37]. These approaches will facilitate the development of effective conservation strategies and improve the management of genetic diversity in captive and wild populations.

Molecular markers have been useful for determining kinship coefficients, especially when detailed pedigree information is unavailable. The combination of multiple methods to identify parentage and classify kinship between individuals (e.g., parent-offspring, full-siblings, half-siblings, and unrelated) between individuals could improve the estimation of kinship coefficients [38]. However, the use of combined methods typically necessitates gathering observational data to construct pedigrees, which can be challenging to obtain for wild species. To allow precise estimation of marker-estimated kinship coefficients, it is recommended to have many marker loci (>200 loci) [22]. The use of genomic kinship estimators, such as the run of homozygosity, produced outcomes comparable to those of traditional pedigree-based coefficients in domestic animals, as demonstrated by Dadousis *et al*. [37]. In the present study, we examined the available mtSNPs for managing captive Asian elephants and emphasized the loci that were inadequate in both quantitative and qualitative (i.e., low Ho) aspects. This indicated the necessity for a larger panel of nuclear SNPs to facilitate the management of Asian elephant populations.

The findings of this study have significant practical applications for the management of captive Asian elephant populations. The observed deficit in heterozygosity and high marker-estimated kinship coefficients underscore the need for more informed breeding protocols to minimize inbreeding and maintain genetic diversity. For example, pairing individuals with lower estimated kinship coefficients based on genomic data can help reduce the risk of inbreeding [26]. In addition, routine genetic monitoring using nuclear SNPs and broader genomic datasets should be implemented to detect and manage genetic bottlenecks and optimize the long-term sustainability of the population. These strategies could be extended to other captive populations facing similar challenges.

## CONCLUSION

This study assessed the genetic diversity and kinship relationships of a captive Asian elephant population at EK, Thailand, using mtSNP markers. The population exhibited a balanced sex ratio (0.97:1) and genetic diversity characterized by moderate levels of heterozygosity (H_o_ = 0.4451, H_e_ = 0.5278). However, deviations from HWE in two loci highlight limitations in the marker panel. The significant discrepancy between pedigree-based (K_ped_ = 0.0032) and marker-based (K_mark_ = 0.4201) kinship coefficients, alongside their weak negative correlation (r = −0.28, p < 0.05), revealed the restricted resolution of the current mtSNP markers for precise kinship estimation.

This study is among the first to evaluate mtSNP markers in the management of genetic diversity in captive Asian elephants in Thailand. This study offers valuable insights into the demographic structure and genetic health of the EK population, providing a foundation for more informed breeding strategies and long-term genetic monitoring. However, reliance on mtSNPs limits the scope of kinship analysis due to maternal inheritance and low marker resolution. Potential inaccuracies in pedigree data, particularly founder assumptions, may have further contributed to the underestimation of pedigree-based kinship coefficients. The absence of nuclear SNPs or microsatellite data limited the ability to perform a more comprehensive genetic analysis.

Future research should focus on expanding the molecular marker panel to include nuclear SNPs or microsatellites to improve the resolution of genetic diversity and kinship estimates. Collaborative studies with other captive elephant populations would help validate these findings and improve our understanding of genetic trends across populations. Longitudinal studies assessing genetic changes over multiple generations and the integration of advanced genomic techniques, such as whole-genome sequencing, could further refine conservation approaches. These efforts will help ensure the genetic health and sustainability of captive Asian elephant populations.

## DATA AVAILABILITY

Supplementary data can be available from the corresponding author upon a reasonable request.

## AUTHORS’ CONTRIBUTIONS

CC and TP: Study conception and design. CC, NB, KP, PC, and NM: Collected and organized the identification certificate data from the EK and constructed pedigree-based pedigree. TP and BS: Database management and statistical analyses of the demographic data. TP, KP, PC, and NM: Analyzed and interpreted the genetic data from identification certificates. TP: Drafted and revised the manuscript. All authors have read and approved the final manuscript.

## References

[1] Krause D.J, Bonin C.A, Goebel M.E, Reiss C.S, Watters G.M (2022). The rapid population collapse of a key marine predator in the Northern Antarctic Peninsula endangers genetic diversity and resilience to climate change. Front. Mar. Sci.

[2] Bansiddhi P, Brown J.L, Thitaram C, Punyapornwithaya V, Nganvongpanit K (2020). Elephant tourism in Thailand:A review of animal welfare practices and needs. J. Appl. Anim. Welf. Sci.

[3] Bansiddhi P, Brown J.L, Thitaram C, Punyapornwithaya V, Somgird C, Edwards K.L, Nganvongpanit K (2018). Changing trends in elephant camp management in Northern Thailand and implications for welfare. PeerJ.

[4] Williams C, Tiwari S, Goswami V, De Silva S, Kumar A, Baskaran N, Yoganand K, Menon V (2020). Elephas maximus. The IUCN Red List of Threatened Species 2020.

[5] Suwattana D, Koykul W, Jirasupphachok J, Kanchanapangka S (2007). Microsatellite polymorphism and parentage control in Thai domestic elephants (*Elephas maximus*). Thai J. Vet. Med.

[6] Thitaram C, Thongtip N, Somgird C, Colenbrander B, van Boxtel D.C.J, van Steenbeek F, Lenstra J.A (2008). Evaluation and selection of microsatellite markers for an identification and parentage test of Asian elephants (*Elephas maximus*). Conserv. Genet.

[7] Thitaram C, Somgird C, Mahasawangkul S, Angkavanich T, Roongsri R, Thongtip N, Colenbrander B, van Steenbeek F.G, Lenstra J.A (2010). Genetic assessment of captive elephant (*Elephas maximus*) populations in Thailand. Conserv. Genet.

[8] Hauser L, Baird M, Hilborn R, Seeb L.W, Seeb J.E (2011). An empirical comparison of SNPs and microsatellites for parentage and kinship assignment in a wild sockeye salmon (*Oncorhynchus nerka*) population. Mol. Ecol. Resour.

[9] Kitpipit T, Thanakiatkrai P, Penchart K, Ouithavon K, Satasook C, Linacre A (2016). Ivory species identification using electrophoresis-based techniques. Electrophoresis.

[10] Kitpipit T, Thongjued K, Penchart K, Ouithavon K, Chotigeat W (2017). Mini-SNaPshot multiplex assays authenticate elephant ivory and simultaneously identify the species origin. Forensic Sci. Int. Genet.

[11] Cardinali I, Tancredi D, Lancioni H (2023). The revolution of animal genomics in forensic sciences. Int. J. Mol. Sci.

[12] Konrad A, Thompson O, Waterston R.H, Moerman D.G, Keightley P.D, Bergthorsson U, Katju V (2017). Mitochondrial mutation rate, spectrum and heteroplasmy in *Caenorhabditis elegans* spontaneous mutation accumulation lines of differing population size. Mol. Biol. Evol.

[13] Charlesworth D, Willis J.H (2009). The genetics of inbreeding depression. Nat. Rev. Genet.

[14] Lozada-Soto E.A, Maltecca C, Lu D, Miller S, Cole J.B, Tiezzi F (2021). Trends in genetic diversity and the effect of inbreeding in American Angus cattle under genomic selection. Genet. Sel. Evol.

[15] Cortes-Hernández J.G, Ruiz-López F.J, Vásquez-Peláez C.G, García-Ruiz A (2022). Runs of homocigosity and its association with productive traits in Mexican Holstein cattle. PLoS One.

[16] Neaves L.E, Eales J, Whitlock R, Hollingsworth P.M, Burke T, Pullin A.S (2015). The fitness consequences of inbreeding in natural populations and their implications for species conservation - a systematic map. Environ. Evid.

[17] Chakraborty S, Boominathan D, Desai A.A, Vidya T.N.C (2014). Using genetic analysis to estimate population size, sex ratio, and social organization in an Asian elephant population in conflict with humans in Alur, Southern India. Conserv. Genet.

[18] Keerthipriya P, Nandini S, Vidya T.N.C (2021). Effects of male age and female presence on male associations in a large, polygynous mammal in Southern India:The Asian elephant. Front. Ecol. Evol.

[19] R Core Team (2013). R:A Language and Environment for Statistical Computing. R Foundation for Statistical Computing, Vienna, Austria.

[20] Raymond M, Rousset F (1995). GENEPOP (version 1.2):Population genetics software for exact tests and ecumenicism. J. Hered.

[21] Royo J.L (2020). Hardy Weinberg equilibrium disturbances in case-control studies lead to non-conclusive results. Cell J.

[22] Eding H, Meuwissen T.H.E (2001). Marker-based estimates of between and within population kinships for the conservation of genetic diversity. J. Anim. Breed. Genet.

[23] Wilcoxon F (1992). Individual comparisons by ranking methods. In:Breakthroughs in Statistics:Methodology and Distribution. Springer, Berlin, Germany.

[24] Ballou J, Lacy R, Ballou J.D, Gilpin M, Foose T (1995). Identifying genetically important individuals for management of genetic diversity in pedigreed populations. Population Management for Survival and Recovery:Analytical Methods and Strategies in Small Population Conservation. Columbia University Press, United States.

[25] Sinnwell J.P, Therneau T.M, Schaid D.J (2014). The kinship2 R package for pedigree data. Hum Hered.

[26] Purisotayo T, Jonsson N.N, Mable B.K, Verreynne F.J (2019). Combining molecular and incomplete observational data to inform management of Southern white rhinoceros (*Ceratotherium simum simum*). Conserv. Genet.

[27] Ochoa A, Storey J.D (2021). Estimating FST and kinship for arbitrary population structures. PLoS Genet.

[28] Comizzoli P, Ottinger M.A (2021). Understanding reproductive aging in wildlife to improve animal conservation and human reproductive health. Front. Cell Dev. Biol.

[29] Serrano-Davies E, Traba J, Arroyo B, Mougeot F, Cusco F, Manosa S, Bota G, Faria N, Villers A, Casas F, Attie C, Devoucoux P, Bretagnolle V, Morales M.B (2023). Biased adult sex ratios in Western Europe populations of little bustard (*Tetrax tetrax*) as a potential warning signal of unbalanced mortalities. Bird Conserv. Int.

[30] Donald P.F (2007). Adult sex ratios in wild bird populations. IBIS.

[31] Hays G.C, Mazaris A.D, Schofield G, Laloë J.O (2017). Population viability at extreme sex-ratio skews produced by temperature-dependent sex determination. Proc. Biol. Sci.

[32] Goudet J, Kay T, Weir B.S (2018). How to estimate kinship. Mol. Ecol.

[33] Vidya T.N.C, Fernando P, Melnick D.J, Sukumar R (2005). Population genetic structure and conservation of Asian elephants (*Elephas maximus*) across India. Anim. Conserv.

[34] Comstock K.E, Wasser S.K, Ostrander E.A (2000). Polymorphic microsatellite DNA loci identified in the African elephant (*Loxodonta africana*). Mol. Ecol.

[35] Kongrit C, Siripunkaw C, Brockelman W.Y, Akkarapatumwong V, Wright T.F, Eggert L.S (2008). Isolation and characterization of dinucleotide microsatellite loci in the Asian elephant (*Elephas maximus*). Mol. Ecol. Resour.

[36] Chiao A, Ge J (2024). Determining lineages between individuals with high-density mitochondrial and Y-chromosomal single-nucleotide polymorphisms. Electrophoresis.

[37] Dadousis C, Ablondi M, Cipolat-Gotet C, van Kaam J.T, Finocchiaro R, Marusi M, Cassandro M, Sabbioni A, Summer A (2023). Genomic inbreeding coefficients using imputed genotypes:Assessing differences among SNP panels in Holstein-Friesian dairy cows. Front. Vet. Sci.

[38] Städele V, Vigilant L (2016). Strategies for determining kinship in wild populations using genetic data. Ecol. Evol.

